# Cut Based Method for Comparing Complex Networks

**DOI:** 10.1038/s41598-018-21532-5

**Published:** 2018-03-23

**Authors:** Qun Liu, Zhishan Dong, En Wang

**Affiliations:** 1 Mathematics School and Institute, Jilin University, Changchun, 130012 China; 2 Department of Computer Science and Technology, Jilin University, Changchun, 130012 China

## Abstract

Revealing the underlying similarity of various complex networks has become both a popular and interdisciplinary topic, with a plethora of relevant application domains. The essence of the similarity here is that network features of the same network type are highly similar, while the features of different kinds of networks present low similarity. In this paper, we introduce and explore a new method for comparing various complex networks based on the cut distance. We show correspondence between the cut distance and the similarity of two networks. This correspondence allows us to consider a broad range of complex networks and explicitly compare various networks with high accuracy. Various machine learning technologies such as genetic algorithms, nearest neighbor classification, and model selection are employed during the comparison process. Our cut method is shown to be suited for comparisons of undirected networks and directed networks, as well as weighted networks. In the model selection process, the results demonstrate that our approach outperforms other state-of-the-art methods with respect to accuracy.

## Introduction

Networks, in particular complex networks, appear as domain structures in a wide range of domains^1–5^, including computer science, biology and sociology^6–9^, *etc*. In most real-world cases, networks are directed, in the sense that the edge between two nodes presents not only the connectivity but also the directionality. For example, in a power grid network^10^, electricity flows directly from one node to the other; in a gene regulatory network^11^, one gene transports the transcription of the other gene, which is obviously directed.

**Figure 1:**
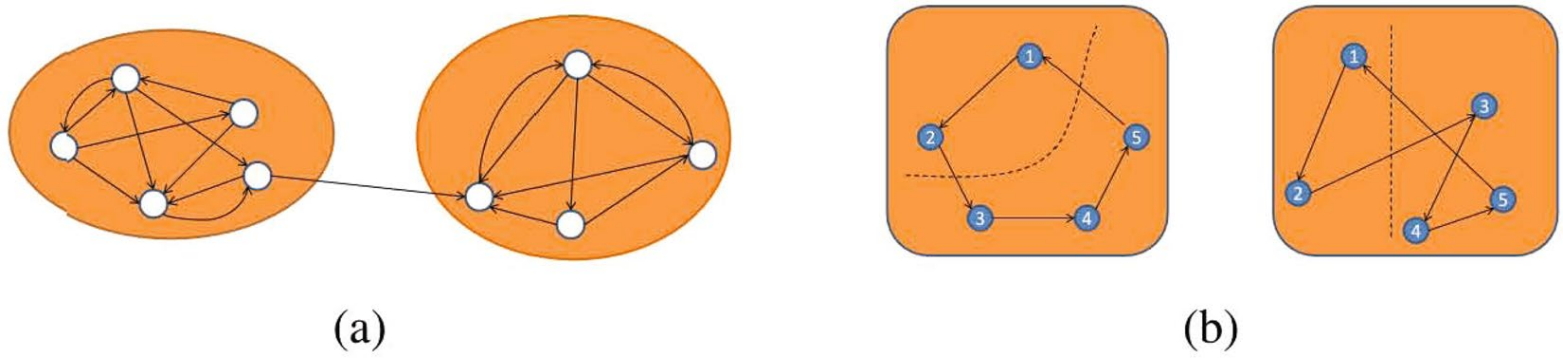
(**a**) Density-based community detection in one directed network. (**b**) Comparing two directed networks. The dot line represents the cut which separates the node set *V* = {1, 2, 3, 4, 5} into two parts, *S* = {1, 2} and *S*^*c*^ = {3, 4, 5}; then, ${e}_{G}(S,{S}^{c})={e}_{G^{\prime} }(S,{S}^{c})=2$. Run all the subsets of *V*, ${d}_{\square }(G,G^{\prime} )=0$.

A central topic in real-world network analysis is the network clustering problem. Because clustering is widely used for studying the structure of various networks, the network clustering problem has attracted much research devoted to statistical and computational issues. Technically, there are two different domains in the network clustering problem. The first domain aims to detect communities in a single network according to a group of regulations (illustrated in Fig. 1(a)), for example, edge density or topology structure, so it is also called the community detection problem; see^12–15^ and the citations therein for more details in this area. On the other hand, the goal of the second domain which is the focus of this paper is to compare a set of diverse networks (illustrated in Fig. 1(b)), regarding them as individual objects^16^. However, the network comparing problem is for the case of undirected networks, and the research on directed networks is still in its infancy, although it is evident that comparing directed networks has many significant applications in different research regimes.

It is common sense that the problem of clustering in directed networks is more challenging than in undirected networks. A common way to deal with directed networks is to simply ignore the edge directionality, in other words, regard the directed networks as undirected and then apply the algorithms for the undirected settings. However, in many situations, this simplistic approach is not satisfactory since the underlying edge directionality needs to be considered (e.g., the directional structure in a citation network between scientific publications). To maintain the information of directionality, in^17^, the author also converts the directed network into an undirected network, but the information about edge directionality is retained via weights on the edges.

The second category of methods is based on spectral graph theory. It is common sense that the adjacency matrix and the Laplacian matrix contain tremendous structural information about graphs^18^. There are many related works and surveys that refer to community detection in directed networks on the basis of spectral methods^19–21^, unfortunately, there are no related works that compare directed networks in the literature.

Among the recent developments in the successful graph comparison algorithms, kernel-based methods^22^ from the machine learning community are the most valuable. The attractiveness of kernel based methods is due to the fact that they can compare networks in polynomial time. In recent years, various graph kernels, which focus on various types of substructures, have been developed, such as subtrees^23^, cycles^24^, random walks^25^, and shortest paths^26^. The other line of existing research has concentrated on graphs in specified structures, such as trees^27^ and strings^28^. Among these algorithms, one can simply and naturally apply the kernel based methods utilized in undirected graphs to directed graphs, just as^29^ did. Unfortunately, there are two unsolved problems in kernel based methods: The first is the problem of choosing the appropriate kernels that can capture network similarity: this is still far from being answered; the other obstacle is that computing any kernel function that is capable of fully recognizing the structure of networks (using subgraph-isomorphism) has been proven to be NP-hard^30^, making it impossible to find efficient kernels.

Apart from the aforementioned methodologies, some research achieves its goals by optimizing various clustering objective functions. A large number of approaches that regard the undirected graph clustering problem as an optimal problem have been presented in recent years, such as modularity^31^ and normalized cut^32^. Recently, some works have tried to extend these measures for directed networks, such as directed vision of modularity^33^ and objective function of weighted cuts in directed networks^34^. In this paper, we develop a framework for comparing directed networks via optimizing an objective function called cut distance^35,36^.

The starting point in this paper is similar to that of ^34^: In^34^, the authors utilized the cut-based method to solve the community detection problem in one directed network; our focus here is to provide a network comparison approach based on the cut distance. In this framework, we will explore and verify this novel network comparison approach via machine learning technologies such as genetic algorithms and model selection. Our cut-based approach to comparing various artificial networks, as well as chemical molecule networks and wild female African elephant dominance networks in the real world, proved to be well suited for comparing undirected networks, directed networks and weighted networks. The corresponding simulations of the model selection process show that our approach works well in comparing directed networks with much higher accuracy than other state-of-the-art methods.

## Results

To illustrate that our framework has strongly mathematical guarantees, in this section, we provide the basic terminology notations that are frequently used in this paper. Although these notations show some differences between dense graphs and sparse graphs, they have no effect on our task, so we only use the terminology for dense graphs; there is no difference if you prefer to use those of sparse settings.

### Graph theory

A graph *G* = (*V*(*G*), *E*(*G*)) consists of a set of nodes *V*(*G*) and a set of edges *E*(*G*) that connect pairs of nodes. In a directed graph *G* = (*V*(*G*), *E*(*G*)), every edge (*i*, *j*) ∈ *E*(*G*) represents a connection from node *i* to *j*. An undirected graph can be seen as a directed graph where if edge (*i*, *j*) ∈ *E*(*G*), then edge (*j*, *i*) ∈ *E*(*G*) as well. We also need some functions to describe the neighbors of a node *V* in a graph *G*: *δ*_+_(*v*) = {(*v*,*u*) ∈ *E*(*G*)} and *δ*_−_(*v*) = {(*u*,*v*) ∈ *E*(*G*)}. Here, *δ*_+_(*v*) and *δ*_−_(*v*) are called the outdegree and indegree of a node *v*, respectively. Furthermore, the maximal outdegree and indegree are denoted by Δ^+^ = max{|*δ*_+_(*v*)|, *v* ∈ *V*(*G*)} and Δ^−^ = max{|*δ*_−_(*v*)|, *v* ∈ *V*(*G*)}, respectively.

In a weighted graph *G*, for each subset *S*,*T*⊂*V*(*G*), define

1 $${e}_{G}(S,T)=\sum _{i\in S,j\in T}{\beta }_{ij}(G),$$

where *β*_*ij*_(*G*) is the associated edge weight for edge *ij*; i.e., when *G* is unweighted, *e*_*G*_(*S*, *T*) is the number of edges with one end in *S* and the other in *T* (illustrated in Fig. 1(b)).

Homomorphism density is a crucial definition in our framework:

#### Definition 1:

*Let F be simple graphs, and define* hom(*F*, *G*) *as the number of homomorphisms from F to G; i.e., the number of adjacency preserving maps V(F) → V(G), and the homomorphism density of F in G is*

2 $$t(F,G)=\frac{1}{{|V(G)|}^{V(F)}}{\rm{\hom }}\,(F,G).$$

*Here*, hom(*F*, *G*) *can be computed via*

3 $${\rm{\hom }}(F,G)=\sum _{\varphi :V(F)\to V(G)}\prod _{i,j\in E(F)}{\beta }_{\varphi (i),\varphi (j)}(G),$$

where the sum runs over all maps from *V*(*F*) to *V*(*G*).

### Cut distance

In this paper, our framework is based on the cut distance (or rectangle distance) proposed by Frieze and Kannan^37^; we strongly recommend the celebrated works^35–39^ if readers are interested in mathematical settings. The following is the explicit mathematical definition of the cut distance. First, we illustrate the cut distance between two graphs with the same set of nodes *V*; the schematic illustration for unweighted graphs can be seen in Fig. 1(b).

#### Definition 2:

*For two graphs G and G′ with the same set of nodes V*,

4 $${d}_{\square }(G,G^{\prime} )=\mathop{{\rm{\max }}}\limits_{S\subset V}\frac{1}{{|V|}^{2}}|{e}_{G}(S,{S}^{c})-{e}_{G^{\prime} }(S,{S}^{c})|,$$

*where S*^*c*^ = *V*\*S*.

According to^35^, to calculate the cut distance between two labeled graphs *G* and *G*′ on *n* and *n*′ nodes, one can compute the cut distance between two new “blow-up graphs” *G*[*n*′] and *G*′[*n*]. The k-fold blow-up of a graph *G* is the graph *G*[*k*] obtained from *G* by replacing each node by *k* independent nodes, and connecting two new nodes if and only if their originals were connected. If *G* is weighted, we define *G*[*k*] to be the graph on *nk* nodes labeled by pairs *iu*, *i* ∈ *V*(*G*),*u* = 1, …, *k*, with edge weights *β*_*iu*,*jv*_(*G*[*k*]) = *β*_*ij*_(*G*). Note that when *n*′/*n* is an integer, it is sufficient to blow-up graph *G* to *G*[*n*′/*n*].

The relationship between the cut distance and homomorphism density is from the following core theorem:^35^

#### Theorem:

*Let G and G*′ *be two graphs; if F is a simple graph with m edges, then*

5 $$|t(F,G)-t(F,G^{\prime} )|\le 4|E(F)|{d}_{\square }(G,G^{\prime} ).$$

Heuristically, if the cut distance between two graphs is small enough, then the homomorphism densities of these two graphs must have similar values for arbitrary small “structure” *F*; in other words, these two graphs must have very similar structures. This is the theoretical basis for our framework.

### Cut distance measures conventional artificial networks

In this subsection, two illustrative examples are utilized to show that the cut distance is very well suited for comparing conventional artificial networks—Erdös-Rényi graphs^40^ with different parameters. Intuitively, two undirected Erdös-Rényi graphs with the same connection probability *p* will have similar topology structures, and the scale of the nodes of these two graphs contribute little to their dissimilarity. In our experiment, we utilize *ER*(*n*, *p*) to represent an undirected Erdös-Rényi instance with *n* nodes, and each unordered pair (*i*, *j*), *i*, *j* ∈ [*n*] is presented by an edge with connection probability *p*, independently of all the other edges. With respect to the directed case *ER*(*n*, *p*_1_, *p*_2_), each pair (*i*, *j*), if *i* < *j*, is presented by a directed edge from *i* to *j* with probability *p*_1_, and a directed edge from *j* to *i* with probability *p*_2_. Unlike the undirected case, two directed Erdös-Rényi graphs, for example, *ER*(*n*, *p*_1_, *p*_2_) and *ER*(*n*, *p*_2_, *p*_1_), will exhibit dissimilarity due to the directionality within edges.

**Figure 2:**
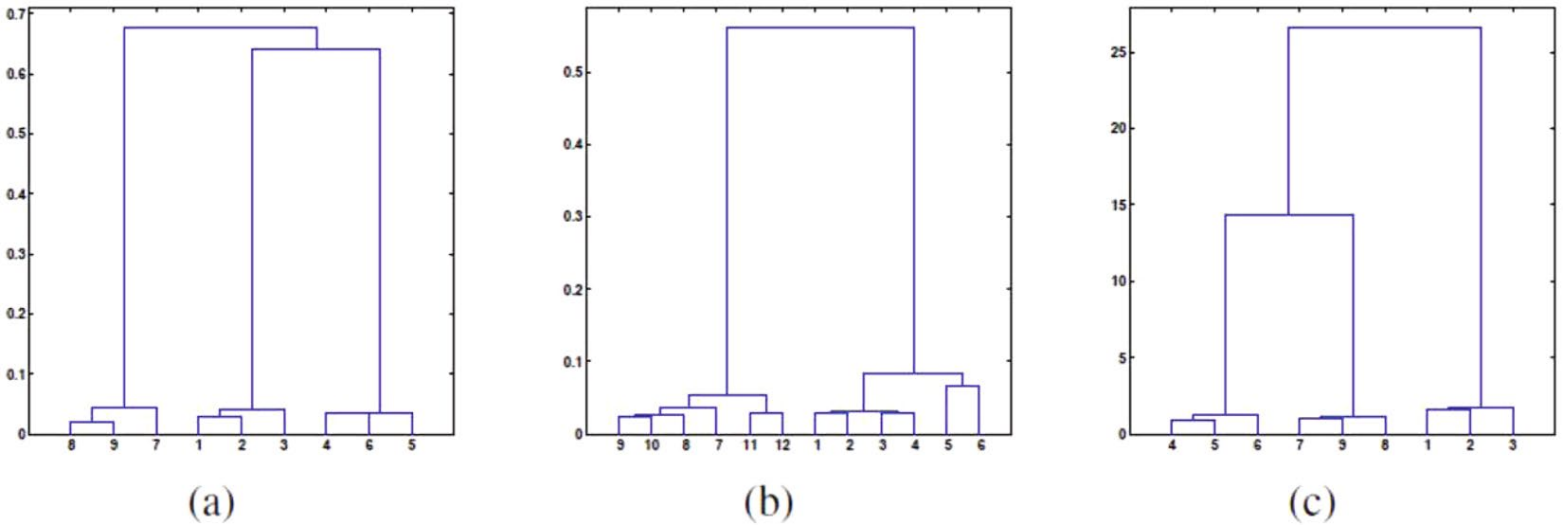
Dendrogram plots for various networks. The labels in the x axis represent the corresponding instances in Tables 1–3 respectively. (**a**) represents the clustering process for undirected Erdös-Rényi graphs. (**b**) represents the clustering process for directed Erdös-Rényi graphs. (**c**) represents the clustering process for directed weighted Erdös-Rényi graphs.

In Table 1, nine different independent undirected Erdös-Rényi graphs with different graphic parameters are generated: For convenience, we utilize the notation (*n*, *p*) to represent an undirected Erdös-Rényi instance with the number of nodes *n* and connection probability *p*. As seen from Table 1 and the corresponding cluster dendrogram in Fig. 2(a), different instances with the same connection probability *p* are well clustered, and the scale of the network contributes little to the clustering process.

**Table 1:** The cut distance matrix for directed Erdös-Rényi graphs with different number of nodes and parameters.

D	(50,0.2)	(100,0.2)	(100,0.2)	(50,0.5)	(100,0.5)	(100,0.5)	(50,0.8)	(100,0.8)	(100,0.8)
(50,0.2)	0	0.0125	0.0138	0.2268	0.2173	0.2266	0.4412	0.4589	0.4600
(100,0.2)	0.0125	0	0.0102	0.2189	0.2172	0.2334	0.4511	0.4643	0.4757
(100,0.2)	0.0138	0.0102	0	0.2406	0.2374	0.2308	0.4713	0.4760	0.4678
(50,0.5)	0.2268	0.2189	0.2406	0	0.0172	0.0156	0.2548	0.2240	0.2305
(100,0.5)	0.2173	0.2172	0.2374	0.0172	0	0.0147	0.2419	0.2395	0.2390
(100,0.5)	0.2266	0.2334	0.2308	0.0156	0.0147	0	0.2360	0.2250	0.2266
(50,0.8)	0.4412	0.4511	0.4713	0.2548	0.2419	0.2360	0	0.0135	0.0127
(100,0.8)	0.4589	0.4643	0.4760	0.2240	0.2395	0.2250	0.0135	0	0.0101
(100,0.8)	0.4600	0.4757	0.4678	0.2305	0.2390	0.2266	0.0127	0.0101	0

In the directed case, we generated 12 independent directed Erdös-Rényi graphs with different parameters. Similarly, we use notation (*n*, *p*_1_, *p*_2_) to represent a directed Erdös-Rényi instance *ER*(*n*, *p*_1_, *p*_2_). From Table 2 and the associated dendrogram plot in Fig. 2(b), it is plausible to cluster these 12 directed graphs into 4 groups: (1, 2, 3, 4), (5, 6), (7, 8, 9, 10), (11, 12). These results show that the cut-distance is a suitable measure to cluster directed graphs, and this simple implementation is also in accordance with our prediction.

**Table 2:** The cut distance matrix for directed Erdös-Rényi graphs with different number of nodes and parameters.

D	(50,0.2,0.6)	(50,0.2,0.6)	(100,0.2,0.6)	(100,0.2,0.6)	(50,0.6,0.2)	(50,0.6,0.2)	(50,0.5,0.8)	(50,0.5,0.8)	(100,0.5,0.8)	(100,0.5,0.8)	(50,0.8,0.5)	(50,0.8,0.5)
(50,0.2,0.6)	0	0.0141	0.0157	0.0147	0.0392	0.0417	0.1967	0.1902	0.1951	0.1993	0.2090	0.1910
(50,0.2,0.6)	0.0141	0	0.0166	0.0154	0.0431	0.0446	0.1906	0.1956	0.1954	0.1975	0.2093	0.2088
(100,0.2,0.6)	0.0157	0.0166	0	0.0119	0.0467	0.0437	0.1779	0.1917	0.1938	0.2019	0.1997	0.1961
(100,0.2,0.6)	0.0147	0.0202	0.0142	0	0.0467	0.0489	0.1924	0.1966	0.1997	0.1971	0.1868	0.1941
(50,0.6,0.2)	0.0392	0.0431	0.0467	0.0452	0	0.0220	0.1930	0.2152	0.2032	0.2139	0.2134	0.2135
(50,0.6,0.2)	0.0417	0.0446	0.0437	0.0411	0.0220	0	0.1755	0.1815	0.1934	0.1910	0.1926	0.1848
(50,0.5,0.8)	0.1967	0.1906	0.1779	0.1924	0.1930	0.1755	0	0.0177	0.0163	0.0170	0.0319	0.0295
(50,0.5,0.8)	0.1902	0.1956	0.1917	0.1966	0.2152	0.1815	0.0177	0	0.0123	0.0162	0.0314	0.0294
(100,0.5,0.8)	0.1951	0.1954	0.1938	0.1997	0.2032	0.1934	0.0163	0.0123	0	0.0127	0.0296	0.0284
(100,0.5,0.8)	0.1993	0.1975	0.2019	0.1971	0.2139	0.1910	0.0170	0.0162	0.0127	0	0.0269	0.0307
(50,0.8,0.5)	0.2090	0.2093	0.1997	0.1868	0.2134	0.1926	0.0319	0.0314	0.0296	0.0269	0	0.0138
(50,0.8,0.5)	0.1910	0.2088	0.1961	0.1941	0.2135	0.1848	0.0295	0.0294	0.0284	0.0307	0.0138	0

In the following part, we demonstrate that the cut distance is capable of comparing weighted networks. We design the experiment as follows: First, we generate 9 directed Erdös-Rényi graphs with *n* = 100, *p*_1_ = 0.5, *p*_2_ = 0.5, and then in each generated graph, we add each edge weight independently according to a uniform distribution. We denote *ER*([0,*a*]) a weighted graph within which each edge weight is assigned from a uniform distribution *U*[0,*a*]. As shown in Table 3 and the corresponding dendrogram plot in Fig. 2(c), the cut distance between two graphs in which edge weights are assigned from the same uniform distribution is much smaller than that from different distributions. This result demonstrates that our cut distance framework performs well in comparisons of directed weighted networks.

**Table 3:** The cut distance matrix for directed weighted Erdös-Rényi graphs with different edge weights.

D	*ER*([0,100])	*ER*([0,100])	*ER*([0,100])	*ER*([0,50])	*ER*([0,50])	*ER*([0,50])	*ER*([0,25])	*ER*([0,25])	*ER*([0,25])
*ER*([0,100])	0	0.9216	0.9355	9.0712	9.1051	9.9368	14.4134	14.6252	14.3390
*ER*([0,100])	0.9216	0	0.9193	8.7676	9.2203	9.1469	14.3175	14.9862	14.0681
*ER*([0,100])	0.9355	0.9193	0	9.5209	9.7795	10.1581	14.2001	14.8115	15.0536
*ER*([0,50])	9.0712	8.7676	9.5209	0	0.4237	0.4238	5.0383	5.0746	5.2899
*ER*([0,50])	9.1051	9.2203	9.7795	0.4237	0	0.5373	5.0749	4.7845	4.9209
*ER*([0,50])	9.9368	9.1469	10.1581	0.4238	0.5373	0	5.2681	4.7529	5.0814
*ER*([0,25])	14.4134	14.3175	14.2001	5.0383	5.0749	5.2681	0	0.2260	0.2425
*ER*([0,25])	14.6252	14.9862	14.8115	5.0746	4.7845	4.7529	0.2260	0	0.2204
*ER*([0,25])	14.3390	14.0681	15.0536	5.2899	4.9209	5.0814	0.2425	0.2204	0

### Experiments on real networks

#### Chemical molecules networks

We perform experiments on two different well-known undirected real network datasets, namely, MUTAG and PTC^41^. The MUTAG and PTC data sets are used to predict the toxicity of chemical molecules based on a comparison of their three-dimensional structure. The average number of nodes and edges per graph in the MUTAG data set are 17.72 and 38.76, respectively, and 26.70 and 52.06 in the PTC data set. We employ cut distance to measure the similarity of the molecules form these two data sets. In our implementation, we choose the NO 1, 2, 8, and 13 samples from the MUTAG data set and the NO 1, 3, 69, and 81 samples from the PTC data set.

**Figure 3:**
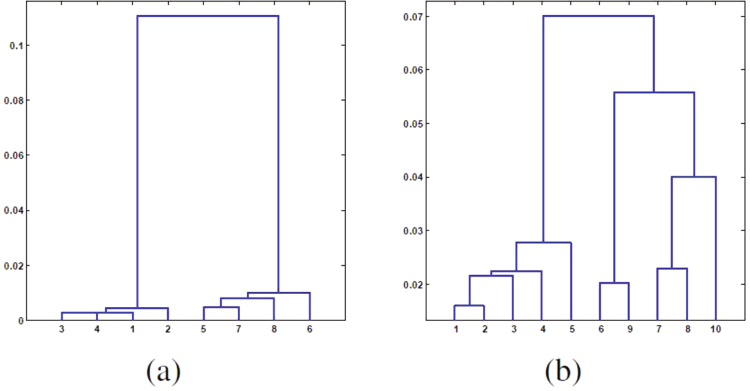
Dendrogram plot for various real networks. The labels in the x axis represent the corresponding instances in Tables 4 and 5. (**a**) represents the clustering process for the MUTAG and PTC networks. (**b**) represents the clustering process for 10 wild female African elephant dominance networks.

A network sample is supposed to be similar to other samples from the same data set; for example, *MUTAG*_1_ is expected to be more similar to *MUTAG*_2_ than to *PTC*_1_. Table 4 exhibits the pairwise cut distances for different MUTAG and PTC network samples. Table 4 and the corresponding dendrogram Fig. 3(a) show that cut distance measurements are consistent with the expectation: Cut distance calculates dissimilarities in such a way that networks of the same type are considered more similar to each other than to networks of different types.

**Table 4:** The cut distance matrix for the MUTAG and PTC data sets.

D	*MUTAG*_1_	*MUTAG*_2_	*MUTAG*_8_	*MUTAG*_13_	*PTC*_1_	*PTC*_3_	*PTC*_69_	*PTC*_81_
*MUTAG*_1_	0	0.0027	0.0017	0.0015	0.0441	0.0405	0.0446	0.0482
*MUTAG*_2_	0.0027	0	0.0029	0.0028	0.0433	0.0395	0.0449	0.0482
*MUTAG*_8_	0.0017	0.0029	0	0.0016	0.0436	0.0415	0.0443	0.0457
*MUTAG*_13_	0.0015	0.0028	0.0016	0	0.0436	0.0407	0.0447	0.0465
*PTC*_1_	0.0441	0.0433	0.0436	0.0436	0	0.0044	0.0031	0.0036
*PTC*_3_	0.0405	0.0395	0.0415	0.0407	0.0044	0	0.0051	0.0077
*PTC*_69_	0.0446	0.0449	0.0443	0.0447	0.0031	0.0051	0	0.0038
*PTC*_81_	0.0482	0.0482	0.0457	0.0465	0.0036	0.0077	0.0038	0

#### Wild female African elephant dominance networks

In this part, 10 wild female African elephant dominance networks are compared. Each dominance network represents the dominance-subordinate relationship between adult females within 5 families each in Amboseli and Tarangire. The original data were collected by Archie *et al*.^42^ in the form of a dominance matrix: Each row in the matrix represents one individual in the family, and the value of the intersection of each row (the aggressor) and column (the loser) shows the number of agonistic interactions won by the aggressor against the loser. In this work, we will use Appleby’s^43^ criteria for re-scoring in the dominance matrix. The criteria are widely used in predicting the linear dominance hierarchies in animal social networks^44^. Individuals that win in each dyad receive a score of 1 in their row at the column position of the subordinate, and losers receive a score of 0. If both individuals win equally or the win-lose relationship is unknown, each receives a score of 0.5 in its row-column position. After re-scoring, we are facing 10 directed weighted networks, where each network represents the dominance hierarchy structure within the wild female African elephant family. It is reasonable to believe that different elephant families in different districts (Amboseli and Tarangire) would have different dominance hierarchy structures, even if they are of the same specie and sexuality. In our experiment, we choose 5 families from Amboseli (AA, PC, EA, EB, OA), and 5 families from Tarangire (T, D, P, PH, SI); the corresponding data are available in Fig. 2 of Archie’s work^42^. As shown in Table 5 and the associated dendrogram plot in Fig. 3(b), three clusters appears: (AA, PC, EA, EB, OA), (T, PH), (D, P, SI). Since the cut distances among T, D, P, PH are very small, i.e., the largest distance among these four families is 0.029, it is plausible to infer that the second cluster (T, PH) and the third cluster (D, P, SI) should be merged into one large cluster. It turns out that these results validate our prediction in addition to showing that our cut distance framework is well suited for comparing real directed weighted networks.

**Table 5:** The cut distance matrix for 10 wild female African elephant dominance networks.

Distance	AA	PC	EA	EB	OA	T	D	P	PH	SI
AA	0	0.0103	0.0141	0.0102	0.0154	0.0546	0.0399	0.0411	0.0617	0.0300
PC	0.0103	0	0.0095	0.0122	0.0143	0.0549	0.0407	0.0395	0.0582	0.0307
EA	0.0141	0.0095	0	0.0142	0.0208	0.0547	0.0481	0.0513	0.0593	0.0361
EB	0.0102	0.0122	0.0142	0	0.0236	0.0624	0.0486	0.0482	0.0596	0.0373
OA	0.0154	0.0143	0.0208	0.0236	0	0.0525	0.0489	0.0417	0.0589	0.0313
T	0.0546	0.0549	0.0547	0.0624	0.0525	0	0.0290	0.0288	0.0107	0.0381
D	0.0399	0.0407	0.0481	0.0486	0.0489	0.0290	0	0.0145	0.0282	0.0165
P	0.0411	0.0395	0.0513	0.0482	0.0417	0.0288	0.0145	0	0.0228	0.0195
PH	0.0617	0.0582	0.0593	0.0596	0.0589	0.0107	0.0282	0.0228	0	0.0417
SI	0.0300	0.0307	0.0361	0.0373	0.0313	0.0381	0.0165	0.0195	0.0417	0

### Compare to other baseline methods

**Figure 4:**
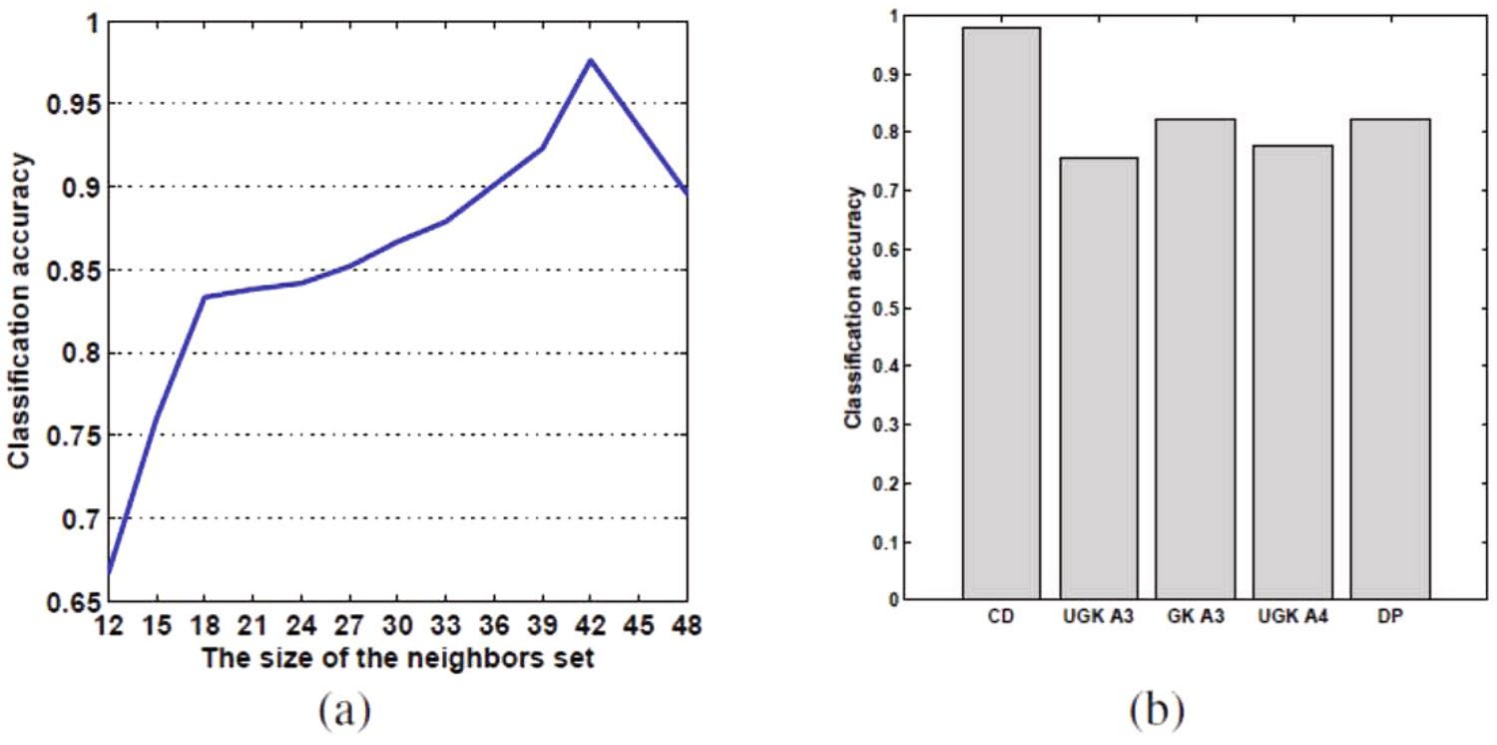
(**a**) The effect of the neighbors set size on the accuracy of the model selection process on the basis of cut distance. (**b**) Classification accuracy based on various comparison methods. CD is the method based on cut distance. UGK Ak denotes the graphlet kernel method via the use of size k undirected graphlets. GK Ak denotes the graphlet kernel method via the use of size k directed graphlets. DP represents the directed product kernel method.

In this subsection, we evaluate the performance of a model selection process on the basis of cut distance and compare it with other baseline methods in terms of prediction accuracy. Our baseline comparators are classic kernel-based methods^29,30,45^, here, we investigate four kinds of kernels: size 3 undirected graphlet kernels, size 3 directed graphlet kernels, size 4 undirected graphlet kernels, and random walks kernels. Figure 4(b) shows the accuracy of the proposed model selection process based on the cut distance along with the baseline methods. As Fig. 4(b) shows, our model selection process based on the cut distance outperforms traditional kernel-based methods with respect to accuracy.

## Methods

### Genetic algorithm

Calculating the explicit cut distance between two graphs is nontrivial, especially for large graphs, since in principle the problem of optimizing over all possible subsets is NP-hard. In this framework, we utilize genetic algorithm^46^ to solve this optimization problem.

**Figure 5:**
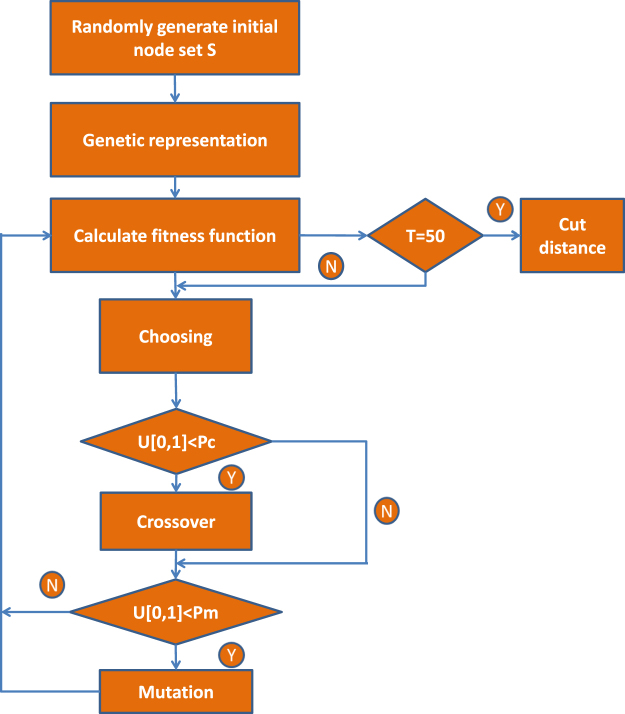
The genetic algorithm for cut distance. Here, *U*[0;1] represents a uniform distribution of interval [0;1].

A genetic algorithm is based on an imitation of the natural selection process; every candidate solution is represented by a chromosome which can be mutated and altered. Typically, solutions are represented by binary strings. In this framework, candidate solutions are different node sets; we transform node sets into a binary string as follows: First, we assume that graphs *G* and *G*′ have the same node set *V*, for each node *i* ∈ *V*,

6 $$\omega (i)=(\begin{array}{cc}1, & i\in S,\\ 0, & {\rm{otherwise}}{\rm{.}}\end{array}$$

so we obtain a length *n* vector that works as the chromosome. In the process of the genetic algorithm, as in the natural selection process in the real world, only those excellent chromosomes remain over the long-time evolution, so we can obtain an optimizing result that approximates the explicit cut distance after the iterations. The related procedure of the genetic algorithm for cut distance can be seen in Fig. 5. In our framework, its fitness would then be given by the corresponding sum—which we are trying to maximize and the set *S* finally evolves, which results in the cut distance defined in Eq. (4).

For the case where *G* and *G*′ have a different number of nodes, by constructing two new blow-up graphs ${G}_{new},{G^{\prime} }_{new}$ with the same node set, the cut distance can then be obtained by computing the distance between these two new blow-up graphs.

### Model selection

The Model selection process is based on a network distance metric that can separate networks among different kinds of models. In this subsection, we give a description of the model selection process.

**Figure 6:**
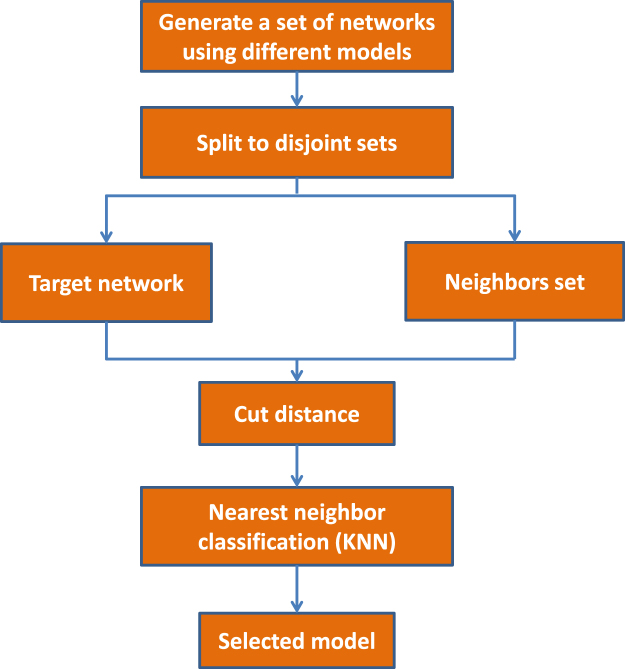
The proposed methodology for model selection.

The corresponding model selection process can be seen in Fig. 6. In the first step, we generate a set of networks using various network models; each network instance is labeled by its corresponding model; *i.e*., if we generate two types of networks, the instances of the first type are labeled 1, and the other instances are labeled 2. In the second step, choose one network instance as the target network; the other instances are regarded as the neighbors set; then, the target network is classified by a majority vote of its neighbors, with the target network being assigned to the class most common among its k nearest neighbors. If *k* = 1, the target network is simply assigned to the class of that single nearest neighbor. Run the second step until all the instances are classified. In essence, this procedure is the classic *k* nearest neighbor classification algorithm (KNN). The corresponding classification accuracy on the basis of a distance *d* is

7 $$accuracy(d)={\mathbb{P}}(knnclass(i)=trueclass(i\mathrm{);\ }i\in [n]),$$

and here, *n* is the number of generated networks.

### Implementation details

Recall that in the first step of the genetic algorithm, each subset *S* is represented via a length *n* chromosome. In the following steps of the genetic algorithm, a new generation of the population is produced by using the crossover and mutation operations. The excellent (more fitting) genes from the parent chromosomes are inherited by descendants through crossover operation with probability *P*_*c*_ = 0.7. To maintain diversity, the mutation operation is utilized by changing each gene in a chromosome into an adverse gene (i.e., 1 → 0 or 0 → 1) with probability *P*_*m*_ = 0.16. The maximum of iterations for the genetic algorithm is set to *T* = 50, and then the local optimal solution for the cut distance is the maximum of the 50 iterations.

In our implementation, the model selection is performed based on the KNN algorithm with *k* = 1; in other words, each target network is assigned to the class of that nearest neighbor. We design the model selection process with 420 instances; that is, 420 different artificial networks with randomly chosen parameters are synthesized by means of the three described models (140 instances per model). Our evaluation includes 10 rounds. In each round, 10% of these generated instances are randomly averagely selected; i.e., 14 instances for each model. These networks are used either in the neighbor set or as the target network (see Fig. 6). Each round includes 42 iterations; in each iteration, one instance out of the 42 directed artificial networks is chosen as the target network, and the remaining instances are utilized as the neighbors set. Accuracy is calculated as its average score in different rounds and iterations. In practice, as Fig. 4(a) shows, the accuracy of this model selection process increases with the size of the neighbors set and reaches a maximum value with 41 networks in the neighbors set. The experiment shows that having more than 41 networks in the neighbors results in no improvement in the accuracy of the model selection.

## Discussion

In this paper, we develop a framework for comparing networks on the basis of cut distance. This framework provides sufficient mathematical guarantees for comparing networks with high accuracy. In practice, we find our approach is suited for comparing undirected networks and works well on directed networks. Furthermore, on the basis of cut distance, we design a model selection process to classify various directed networks. More precisely, the major contributions include:

- Develop a novel network comparison framework on the basis of cut distance.
- Utilize genetic algorithm to evaluate the cut distance between two arbitrary networks.
- Compare various complex networks on the basis of cut distance.
- Design a model selection process to classify various directed networks.

Since in our framework, the cut distance between two graphs is evaluated by the genetic algorithm, the performance of our framework depends on the efficiency of the genetic algorithm. However, as is widely known, as the search space increases, the efficiency of the genetic algorithm decreases. As our experience shows, our framework works well for comparing networks around thousands of nodes, but it is not capable of completing the task of comparing very large networks, such as networks with millions of nodes. To address this problem, one may try using other fast optimal algorithms to reevaluate the cut distance.

As noted in our research, the cut distance we investigate is only capable of comparing unlabeled networks. To break this ceiling, in future work, we will develop a network comparison method based on a new cut distance. First, we have a look at the definition:

### Definition

*Given two graphs G*_1_ *and G*_2_, *then the new cut distance is*

8 $${\delta }_{\square }({G}_{1},{G}_{2})=\mathop{{\rm{\min }}}\limits_{G\cong {G}_{2}}{d}_{\square }({G}_{1},G)\mathrm{.}$$

*Here we write G* ≅ *G*′ *if G and G*′ *are isomorphic, i.e., if G*′ *can be obtained from G by a relabeling of its nodes*.

The reason we want to utilize this new cut distance can be summarized as follows:

- The new cut distance ${\delta }_{\square }$ can be utilized in comparing unlabeled networks, while the cut distance ${d}_{\square }$ can only deal with labeled networks.
- In^35^, the authors developed a rigorous mathematical theory based on the cut distance ${\delta }_{\square }$, and we can have better mathematical guarantees than those of ${d}_{\square }$.

The main obstacle is to evaluate the cut distance ${\delta }_{\square }$; as far as we know, the graph isomorphic problem is NP-hard in computer science. Even if it is NP-hard, we strongly believe one may achieve this goal by optimizing various clustering objective functions.

### Artificial networks data description

The utilized network generation models and the synthesized graphs of the artificial networks data set are described in the following:

#### Directed Erdös-Rényi model

This model generates random graphs with two specified density *p*_1_ and *p*_2_. In our implementation, *p*_1_ is 0.08, and the other density parameter is chosen randomly from the range 0.02 ≤ *p*_2_ ≤ 0.1.

#### Directed preferential attachment model

In this model, we begin with an initial directed graph *G*(*t*_0_) with *t*_0_ edges. For *t* ≥ *t*_0_, we generate directed graph *G*(*t*) according to the following three rules^47^.

(A) With probability *α*, add a new node *v* together with *m*_1_ edges from *v* to *m*_1_ existing nodes which are chosen according to ${D}_{i}^{(in)}(t)+{\delta }_{in}$.
(B) With probability *β*, *m*_2_ new edges are added between the existing nodes ${v}_{1},\cdots ,{v}_{{m}_{2}}$ and ${w}_{1},\cdots ,{w}_{{m}_{2}}$, where ${v}_{1},\cdots \mathrm{.}{v}_{{m}_{2}}$ and ${w}_{1},\cdots ,{w}_{{m}_{2}}$ are chosen independently, *v*_*i*_ is selected according to ${D}_{i}^{(in)}(t)+{\delta }_{in}$, and *w*_*i*_ is selected according to ${D}_{i}^{(out)}(t)+{\delta }_{out}$.
(C) With probability *γ*, we add a new node *w* and *m*_3_ edges from *m*_3_ existing nodes to *w*,where the chosen nodes are selected according to ${D}_{i}^{(out)}(t)+{\delta }_{out}$.

In our implementation, we fix *α* = 0.3, *β* = 0.5 and *γ* = 0.2; *δ*_*in*_ and *δ*_*out*_ are fixed as 1 and 2, respectively; the edge parameters *m*_1_ and *m*_2_ are fixed as 1 and 2, respectively; the range of edge parameter *m*_3_ in our evaluations is 1 ≤ *m*_3_ ≤ 5.

#### Directed configuration model

This model is configured by two degree sequences—in-degree and out-degree. In our implementation, each element of the in-degree and out-degree sequence is chosen randomly from the set {2, 3, 4, 5, 6} with identical probability 1/5^48^.

### Baselines

For comparison, we briefly introduce the traditional kernel-based algorithms.

#### Graphlet kernels

Literally, graphlets are the subgraphs with *k* nodes. Let ${\mathbb{G}}=\{graphlet\mathrm{(1),}\,\cdots ,\,graphlet({N}_{k})\}$ be the set of size-k graphlets and *G* be a graph of size *n*. Define a vector *f*_*G*_ of length *N*_*k*_ whose i-th component corresponds to the frequency of occurrence of *graphlet(i)* in *G*. *f*_*G*_ is called the k-spectrum of *G*. In order to account for differences in size of the graphs, we normalize the counts to probability vectors^45^:

9 $${D}_{G}=\frac{1}{|all\,graphlets\,in\,G|}{f}_{G}\mathrm{.}$$

Given two graphs *G* and *G*′ of size *n* ≥ *k*, the graphlet kernel *k*_*g*_ is then defined as

10 $${k}_{g}(G,G^{\prime} )={D}_{G}^{T}{D}_{G}.$$

In our experimental setting, we compute all undirected size-3, size-4 graphlet kernels and directed size-3 graphlet kernel^29^.

#### Direct product kernel

Let *G*_1_(*V*_1_,*E*_1_),*G*_2_(*V*_2_,*E*_2_) be two graphs; let *A*_×_ denote the adjacency matrix of their direct product *G*_1_ × *G*_2_; here, *G*_1_ × *G*_2_ is a graph with vertex set^30^:

11 $${V}_{\times }=\{({v}_{i},{v^{\prime} }_{r}):{v}_{i}\in {V}_{1},{v^{\prime} }_{r}\in {V}_{2}\},$$

and edge set

12 $${E}_{\times }=\{(({v}_{i},{v^{\prime} }_{r}),({v}_{j},{v^{\prime} }_{s})):({v}_{i},{v}_{j})\in {E}_{1}\wedge ({v^{\prime} }_{r},{v^{\prime} }_{s})\in {E}_{2}\}\mathrm{.}$$

In other words, *G*_1_ × *G*_2_ is a graph over pairs of vertices from *G*_1_ and *G*_2_, and two vertices in *G*_1_ × *G*_2_ are neighbors if and only if the corresponding vertices in *G*_1_ and *G*_2_ are both neighbors. With a sequence of weights *λ* = *λ*_0_,*λ*_1_, … (${\lambda }_{i}\in {\mathbb{R}}$; *λ*_*i*_ ≥ 0 for all $i\in {\mathbb{N}}$) the direct product kernel is defined as

13 $${k}_{\times }({G}_{1},{G}_{2})=\sum _{i,j\mathrm{=1}}^{|{V}_{\times }|}\,{[\sum _{n\mathrm{=0}}^{\infty }{\lambda }_{n}{A}_{\times }^{n}]}_{ij}$$

if the limit exists.

In our implementation, to make sure the kernel Eq. (13) exists, we let {*λ*_*i*_} be a geometric series, that is *λ*_*i*_ = *γ*^*i*^; here, $\gamma =\frac{1}{a+1}$, where *a* = min{Δ^+^(*G*_1_ × *G*_2_), Δ^−^(*G*_1_ × *G*_2_)}.
